# Do European hospitals have quality and safety governance systems and structures in place?

**DOI:** 10.1136/qshc.2008.029306

**Published:** 2009-01-26

**Authors:** C Shaw, B Kutryba, H Crisp, P Vallejo, R Suñol

**Affiliations:** 1 European Society for Quality in Healthcare, Limerick, Ireland; 2 National Centre for Quality Assessment in Health Care (NCQA), Krakow, Poland; 3 Healthcare Accreditation and Quality Unit, CHKS, London, UK; 4 Avedis Donabedian Institute, Autonomous University of Barcelona, and CIBER Epidemiology and Public Health (CIBERESP), Barcelona, Spain

## Abstract

Internal systems for quality and safety were assessed in 89 hospitals in six European states, by external teams using standardised criteria and procedures, as part of the Methods of Assessing Response to Quality Improvement Strategies (MARQuIS) project. The assessments were made primarily to identify the current use of quality management systems in the sample hospitals, and also to demonstrate a potential tool for comparable assessment of hospitals in general. The large majority of the hospitals had a formal, documented infrastructure to manage quality and safety, but a significant minority had no designated mission, programme or coordination. In two-thirds of hospitals, the governing body was active in defining policy and programmes for improvement, and received reports on quality, safety and patient satisfaction at least once a year. The brief on-site assessments identified systematic variations, within and between countries, in structures and processes of governance and to document the uptake of best practice. Unacceptable variations in practice could be reduced, to the benefit of consumers and providers, by developing and publishing basic organisational standards relevant to all European states. The simple assessment criteria designed for this project could be developed into a practical tool for self-assessment, peer review or benchmarking of hospitals across national borders. This assessment, combined with explicit, relevant and achievable standards, could provide a vehicle to promote the voluntary uptake of best practice and consistency in quality and safety among hospitals in Europe.

Leadership and organisational culture are widely claimed to be essential ingredients of an effective programme to improve quality and safety. Their prevalence in healthcare organisations is hard to estimate directly, but may be inferred from proxy measures of organisational structure and activity.

## GOVERNANCE AND MANAGEMENT

In North America, the responsibilities of hospital trustees and managers for performance, quality and safety—and the required basic structures and systems—have long been defined. In Canada, for example, a panel on accountability in the voluntary sector specifically identified the role of the governing body to include “responsibility for outcomes, including problems created or not corrected by an organisation or its officials and staff”.^1^ Hospital associations, accreditation programmes and regulators have issued explicit guidance and direction; according to the Joint Commission on the Accreditation of Healthcare Organizations (JCAHO), each hospital should have “governance with ultimate responsibility and legal authority for the safety and quality of care, treatment, and services”.

The report of a study of four Organisation for Economic Co-operation and Development (OECD) countries has commented^2^:

“It was formerly taken for granted that the institutions of professional self-regulation would ensure adequate quality of care. However, as scandals and evidence of medical errors and quality shortfalls have emerged in various OECD countries, doubt has been cast on the ability of those institutions to live up to their responsibilities.”

In eastern Europe, the devolution of hospital responsibilities from central to local government has led, in the absence of effective control mechanisms, to “an accountability vacuum” in the region.^3^ Local bodies are charged with “supervision” of public hospital managers but have only limited defined responsibilities for governance, particularly with respect to accountability for the quality and safety of patient care delivered by the hospitals. In eastern Europe, perhaps comforted by lower levels of litigation and regulation, hospitals have been less regimented than in North America. But increasing legal liability for the safety of staff and patients, increasing public expectations and increasing pressure for harmonisation between countries are demanding more transparency and accountability.

In the UK, the public inquiry into management failures in a provincial teaching hospital identified a chain of root causes from local management up to the Department of Health.^4^ Many of these had existed for a long time in the UK National Health Service (NHS), and many of them still exist in other countries, which could be spared the pain and expense of such public scandal if the lessons were shared. The inquiry set a milestone in the development of healthcare in the UK by leading to clarification in law of the individual and corporate responsibilities of clinicians, managers and governing bodies for the safety of patients, staff and public.

Hospital governance was not the principal focus of the Methods of Assessing Response to Quality Improvement Strategies (MARQuIS) project,^5^ but the data which were gathered by visiting 89 hospitals give an insight into current strengths and opportunities for improvement.

## AIM OF THE ON-SITE EXTERNAL ASSESSMENTS

The primary aim of the study was to assess the relationship between quality improvement strategies and the (self-reported) compliance to quality requirements for cross-border patient care and to get additional information to validate the findings of a preliminary questionnaire.^6^ In practice this meant:

- to compare results from questionnaires with information obtained from different methods and to validate it by “triangulation”;
- to obtain detailed evidence of the institutionalisation of quality and safety, and the use of different strategies, including descriptive variables of hospital structure, process and performance.

The secondary aim was to develop and test a simple tool and procedures that could be used for self-assessment or peer review. There are few published precedents to multinational on-site hospital assessment and comparative reporting. Quantitative data-based studies, such as the Performance Assessment Tool for Quality Improvement in Hospitals (PATH) project,^7^ require standardised definition of indicators but do not use on-site visits for validation. Qualitative standards-based programmes, such as the Health Promoting Hospitals project,^8^ rely on self-assessment rather than external independent observers. Regional, national and international hospital accreditation programmes either do not collect or do not publish comparable results between countries.

It was thus necessary to build on a variety of models in order to design an innovative assessment tool to meet the objectives and constraints of the MARQuIS project.

## METHODS

### Design principles of the on-site assessment

The aim was not to promote change in the quality improvement of the hospitals but to describe them according to criteria which had been defined as important for this research project. For that purpose, the methodology was different from that used for organisational development, such as accreditation. Ideally, the hospitals would have been totally “blind” to the assessments and the criteria to avoid distorting the results, but, in reality, the hospitals had to have advance warning in order to consent to participate, to schedule visits and to share the burden of data collection. The assessment criteria and procedures would have to be relevant, understandable and measurable not only in a range of hospitals (public, private, academic) but also in a variety of political, management and funding systems.

To fit within the available time and funding, and to minimise complexity and observer variation, several principles were adopted:

- Organisation: coordinators for each participating country would arrange the recruitment and cascade training of a cadre of visitors (already experienced as accreditation surveyors), based on training given to all the country coordinators at one joint workshop.
- Assessment teams: visits to selected hospitals would be organised by country coordinators; each team would comprise two members, without interchange of assessors between countries.
- Visit schedule: each would be scheduled over 1.5 days; the primary data collection would take 12 man-hours.
- Hospital preparation: to minimise preparation time for the hospital, there would be no self-assessment.
- Assessment tool: to focus on documentary evidence and observation rather than interview with staff; minimal analysis, interpretation or free text would be required from visitors.
- Assessment procedures: would rely on hospitals to collect documentary evidence before the visit, but avoid direct access to patients, or their personal records; would limit the number of locations visited within the hospital and rely on representative tracers and small sample sizes.

### Hospital assessment tool

#### Principles of content

The criteria were designed to relate to values and policies identified in previous work packages; to be verifiable from documentation, interview or direct observation; and to be based as far as possible on existing, tested external assessment criteria for internal systems.

#### Scope, content and structure

The contents of the preliminary survey questionnaire were designed around the quality dimensions that were adopted for the WHO PATH indicators project: clinical effectiveness, production efficiency, staff orientation, responsive governance, and, especially, safety and patient-centredness. The survey questions were then mapped against the classification of quality strategies and tools published by WHO^9^ and the chapter headings and subheadings of standards for two accreditation programmes (UK Hospital Accreditation Programme^10^ and Joint Commission International^11^). This procedure identified a variety of issues which were not included in the initial questionnaire but were relevant to basic management systems such as for environmental safety, waste management, and radiation. The final version of the tool included more than 60% of the questionnaire but excluded criteria that could not be directly verified by observation.

Iterative analysis and discussion with country coordinators produced a prototype version for testing in two sites (one in Ireland, one in Spain) in October 2006. This included a list, provided in advance to the hospitals, of 88 documents that the visitors needed to access on site to evidence some 90% of the criteria. The documents included minutes, reports, policies and procedures at hospital and department level.

#### Rating system

A five-point scoring system was adopted to differentiate between various stages in the development, use and impact of quality systems. In general, the scale reflected: 

A: exceptional compliance >90%;

B: extensive compliance 66–90%;

C: broad compliance 41–65%;

D: minor compliance 15–40%;

E: negligible compliance <15%. 

The percentages were more as a guide rather than as a statistical value, but helped to describe a non-parametric distribution of variation between hospitals. In some instances, intermediate values were not permitted, such as where a “yes” or “no” answer was required.

#### The final version

Lessons from the test sites (about the validity, reliability and feasibility of the assessment) were combined with feedback from the research partners and the country coordinators to produce a final version. This comprised 233 criteria under nine headings (management, human resources, wards, maternity, medical, surgical, pharmacy, records, environment).

## APPLICATION

### Sample selection

The MARQuIS field test had two phases: the first phase consisted of the assessment of the quality improvement system in a sample of European hospitals using a self-administered questionnaire. The second phase was the on-site external assessment of a sample of 105 of the 389 hospitals that answered the questionnaire.^6^ This sample was selected according to:

- level of maturity of their quality improvement as determined by a selection of questions from the MARQuIS questionnaire (sampling for the external assessment was made from the two extreme quartiles so as to compare most and least mature hospitals);
- number of hospitals per country that had answered the questionnaire (all participating countries had to be represented);
- type of hospital—teaching, university, non-teaching;
- representativeness of the hospitals from the group categorised as “known to deliver cross-border care”.

### Preparation for external assessments

Country coordinators confirmed consent of hospitals to on-site assessments and coordinated schedules and logistics. The visiting team received, 1 week in advance, a basic descriptive profile of the hospital extracted from the questionnaire response, but were completely blind to the detailed responses. Hospitals received a list of required documents in advance, but no other detail of the assessment tool.

The Hospital Programme for Europe (European Hospital and Healthcare Federation, HOPE) was responsible for overall coordination of the site visits and for monitoring the achievement of agreed schedules. HOPE also set up a register of frequently asked questions from teams in the field and a hotline for technical problems on-site.

### Data collection

Data were collected from January to April 2007. Most external assessments took 2 days, even when the schedule was adapted to the size and the number of departments to be assessed in each hospital. About 90% of the criteria were directly assessed based on hospital documentation, 5% on information directly asked of hospital staff and the remaining 5% were directly observed by the surveyors. Surveyors sent a completed report format for each of the hospitals to the country coordinator for checking completeness and consistency before submission to the project coordinator (FAD) for validation and compilation into one general database prior to statistical analysis with SPSS V.15.

## RESULTS

### Actual versus expected participation

Although all the hospitals participating in the MARQuIS field test had originally agreed to participate in the external assessment, some of them later declined. The most common reasons for not participating as reported to the country coordinators were being too busy to handle a 2-day visit (or merely being wearied by an overload of quality assessment), and changes in the management team (current managers were not the ones who had agreed participation and were not interested in the project). Despite assurances to hospitals that the resulting data would remain confidential to the research project, several hospitals were discouraged by anxieties of possible repercussions from participation. Of the initially planned 105 hospitals, 89 were finally included in the external assessments. The distribution of these hospitals by country is shown in table 1.

**Table 1:** Distribution of external assessments by country

Country	Assessments expected	Assessments conducted
Belgium	6	1
Czech Republic	15	15
France	21	18
Ireland	8	6
Poland	15	15
Spain	30	29
The Netherlands	4	0
UK	6	5
Total	105	89

### Findings of the visits

The results relating to ward level, in particular to patient safety and cross-border care, are described in separate papers.^12^ This paper concerns the governance and management of quality and safety at hospital level, primarily the aggregated responses to criteria for general management and human resource management.

### The governing body of the hospital

Hospital mission statements were reviewed to check if they explicitly stated that the hospital was committed to quality and safety. Fifteen hospitals did not have an official published mission, so they could not be included in this analysis. Of the 74 hospitals that had a mission statement, 89% explicitly committed the organisation to quality improvement, whereas 50% committed the organisation to improving safety.

Governing body responsibilities and involvement with the hospitals’ quality improvement system were specifically assessed by checking if they had directly participated in some quality improvement activities during 2006. The degree of extensive or full participation is given in table 2, which shows that in around six out of 10 hospitals the governing body approved an annual programme for improvement, and four out of 10 received formal reports on quality, safety and patient satisfaction surveys.

**Table 2:** Governing body responsibilities

	% A,B*	Total number
Has published a mission statement	83	89
Mission commits to improving quality	89	74
Mission commits to improving safety	50	74
Governing body approved annual programme for improvement	57	89
Governing body received formal reports on quality and safety	43	89
Results of patient satisfaction surveys were formally reported	36	89
Results of surveys of provider or other staff satisfaction were reported	12	89

*% Hospitals extensively (B) or exceptionally (A) compliant with criterion.

Governing bodies without a mission statement received less information on quality and safety than those with such a statement. Those with a statement which specified commitment to quality and safety (three quarters of all hospitals) received more information than those which did not. Almost two-thirds of them approved an annual programme for improvement, half received regular reports on quality and safety, and received reports on surveys of patient experience (table 3).

**Table 3:** Mission related to activity of governing body

Mission statement	Governing body activity	% Yes	Total number
No statement	Approves annual programme	20	15
	Receives reports Q&S	13	15
	Receives reports patient experience	0	15
No commitment	Approves annual programme	38	8
	Receives reports Q&S	25	8
	Receives reports patient experience	38	8
Commitment to	Approves annual programme	67	63
quality, safety	Receives reports Q&S	49	63
	Receives reports patient experience	40	63

Three of the hospitals with a statement committed to quality and safety (Q&S) were excluded from this secondary analysis.

### Management of quality systems

In 73 of the hospitals assessed (82%), there was a committee assigned to quality improvement; two-thirds of these held three or more meetings in the previous year, whereas the remainder met more rarely (if at all) or had not kept any written records (table 4). A documented quality improvement action plan for the hospital was evident in two-thirds hospitals but in 10 of these hospitals the plan had not been reviewed or updated for the past 10 years. There was a representative body of the medical staff that accepted corporate responsibility for the quality of medical care in 67% of the hospitals, but was accountable to the governing body only in half of those 60 hospitals. A similar representative body of the nursing staff accepted corporate responsibility for the quality of nursing care in 75% of the hospitals, and was considered accountable to the governing body in just over half of them.

**Table 4:** Corporate responsibilities for quality

	% A,B	Totalnumber
Quality improvement action plan exists	65	89
A committee is assigned to quality improvement	82	89
The committee met in the past year	66	73
Accountable representatives of the medical staff accept corporate responsibility for the quality of medical care	34	89
Representatives of the nursing staff accept corporate responsibility for the quality of nursing care	42	89

*% Hospitals extensively (B) or exceptionally (A) compliant with criterion.

Most hospitals (78%) had a designated leader of quality improvement directly accountable to the chief executive officer (CEO) (table 5); a few more had a designated staff member responsible for coordinating quality improvement between hospital departments. In three out of 10 hospitals, the job descriptions of clinical specialty directors specifically included responsibility for quality and safety. An individual specialist was identified as responsible for the coordination of resuscitation services and supervision of training within the hospital in 48%.

**Table 5:** Individual responsibilities for quality

	% A,B	Totalnumber
Designated leader of quality improvement is directly accountable to the CEO	78	89
Designated person responsible for coordinating between hospital departments	83	87
Clinical director job descriptions require active support of quality and patient safety in:		
maternity service	29	77
cardiology/medical service	31	81
surgical unit	32	84
An identified specialist physician is responsible for the coordination of resuscitation services and training	48	86

*% Hospitals extensively (B) or exceptionally (A) compliant with criterion.

### Staff training and development

Personnel records were reviewed for medical and nursing staff. The content of medical staff files (copy of documents related to licence, education, experience, and certification) was rated as appropriate (extensively or fully compliant) in 75% of hospitals (table 6). For nursing staff, evidence of being currently registered with the relevant nursing council was rated extensive or full in 81% of hospitals. Records of individual attendance at continuing education programmes were complete in 67% of hospitals, higher for fire training, but lower for basic life support.

**Table 6:** Staff development for quality

	% A,B	Totalnumber
Medical staff records contain documents related to licence, education, experience, and certification	75	87
Performance of individual medical staff is formally reviewed once every three years	27	83
Nursing records include evidence of being currently registered	81	85
Qualified nurses have an annual appraisal and development plan	46	84
Records are kept of individual continuing education	67	88
Documented evidence of staff training in fire response and evacuation procedures	75	88
Documented evidence of staff successfully completing initial or refresher training in basic life support	56	88

*% Hospitals extensively (B) or exceptionally (A) compliant with criterion.

Formal review of individual medical staff once every 3 years to determine their continued competence to provide patient care services was rated extensive or full in a minority (27%) of hospitals. Qualified nurses had an appraisal and development plan documented annually in almost half the hospitals assessed.

## DISCUSSION

**Key messages**

- There is wide variation within and between six European countries in the management of quality and safety in hospitals. In some hospitals, basic systems are lacking; in others they exist but do not function. This should be of concern to patients, providers, funding agencies and governments
- Valuable experience could be shared between hospitals through a voluntary code of European practice, based on the findings of this MARQuIS study, the recommendations of other European research projects, and other authoritative guidance and directives
- Compliance with this common code could be promoted by voluntary self-assessment and peer review, using a measurement tool similar to the one designed for the external assessment of hospitals in this study

### Methods

#### Sampling

The uneven representation of the countries involved in this study inhibits direct correlations with national systems, policy, legislation or culture but represents a spectrum of reality. The number of hospitals visited and the depth of investigation were limited by practicalities of time and money but were sufficient to show wide variations. Whenever possible, if one given hospital declined to participate, another hospital with similar characteristics was selected as a “replacement” in order to mitigate the effect of self-selection.

#### Assessment tool and procedure

Few queries were received from the survey teams while in the field, but interpretation of terms such as “teaching accreditation” and “governing body” needed clarification. Discrimination between quality and safety (in mission statements, policies or committee titles) may have been artificial. Omission of “safety” might result either from local interpretation (safety being a part of quality, thus not addressed separately) or from the relatively low level of awareness about the significance of patient safety as a distinct quality dimension. Although the assessment tool was progressively abbreviated and simplified before it was applied, several teams felt pressed for time to collect the required data. Nevertheless, time constraints led to compromises both in the design and application of the tool, including:

- bias towards the observable: reliance on retrieval and completeness of management records;
- simplicity rather than sophisticated and comprehensive analysis and explanation;
- minimal dialogue with clinical staff and none with patients;
- reliance on representative tracers (such as existence and use of guidelines, indicators, etc.);
- small sample sizes (ward visits, staff interviews, document reviews, etc.).

Interobserver variation within and between countries may have been reduced by initial joint training and written guidelines but may have been enabled by translation of the tool into local language, in cascading of training at national level, and by differing cultural assumptions and local health systems.

### Findings

The large majority of hospitals in this study met common expectations of internal quality systems—planning, leadership, committees, guidelines and activities—but few could demonstrate improved procedures, let alone outcomes. In many hospitals, mission statements, plans, committees and systems were evident only on paper.

#### Governing body

Top-level commitment and leadership are frequently stated requirements to develop an organisational culture for effective improvement in quality and safety. Most governing bodies in this study demonstrated this commitment by setting and monitoring policies and procedures. A small proportion either did not document or were not involved in leading the agenda for quality, safety or patient satisfaction. In four out of 10 hospitals, the governing body had not approved an annual programme or received formal reports on quality, safety or patient satisfaction during the past year.

In several countries, litigation, legislation or public inquiries have made clear that governing bodies may be held liable for failures of systems and individuals within the hospitals which they govern. Even if this legal principle has not yet been established in every European country, governing bodies may be well advised to take an active role in defining, measuring and improving standards in healthcare.

### Management

Although most hospitals have a formal infrastructure to manage quality and safety, a significant minority have no designated coordinator, documented programme or committees. “Quality” committees were documented in all but 20% of hospitals in this study, but one in 10 had not met in the past year.

Professional clinical staff accepted corporate responsibility for the quality of care in most hospitals, but were accountable to the governing body for this in only a minority of hospitals (34% medical, 42% nursing). The principle, long adopted in North America, of at least 3-yearly review of medical staff (in public or private sector, whether employed or not) has clearly not reached European hospitals; 75% have no such system.

The organisation and management of clinical staff are key determinants of hospital quality systems, particularly relating to doctors, and in the private sector. The structured systems of North America, such as medical staff bylaws, credentialling and peer review, deserve attention in Europe to fill the evident gap in professional accountability, self-regulation and clinical governance.

### Leadership

Apparently, in a minority of hospitals, there is no leadership of quality and safety by the professions, the management or the governing body. The raw figures gathered in this study give no insight into why some hospitals appear to show so little interest in issues such as safety, performance or patients’ experiences. Lack of litigation, competition, comparative information or financial incentives may sustain a tradition of perceived security in hospitals.

Corporate responsibility lies primarily with governing bodies of individual hospitals but public sector ministries and private sector regulators must also share the burden of stewardship. Accountability and structures should be embedded in guidance routinely issued by regional and national authorities.

### Context of the MARQuIS project

The project overall focused on the quality requirements for cross-border patients within the European Union, including different quality strategies at a national level and how these have been applied by hospitals in a sample of states. This paper focuses on how the on-site external assessments were carried out and discusses a subset of the resulting findings which relate to hospital governance, and the implications of both for organisational development and harmonisation of hospitals in general.

## CONCLUSION

Despite its limitations this study has generated some cogent questions for professions, managers, governing bodies and regional and national health authorities about the nature of governance and accountability. The familiar trappings of quality systems found in most of the hospitals—mission statement, committees and coordinators—are not consistently effective in assuring professions, managers, governing bodies or patients of quality and safety in their hospitals. And the small proportion of hospitals which have no such systems should be a concern both to their responsible authorities and to the populations they serve. Many of these issues cross national borders but remain the responsibility of individual states. Yet the European vision, including cross-border patient care, medical tourism, market competition and reciprocal recognition of clinical training, demands more clear and consistent definition of the organisation of hospital services and accountability for the duty of care.

Some European Union member states, such as France, Ireland and the UK, have already defined and published organisational standards for hospitals, and established a national body to monitor compliance. Some, such as Spain and Italy, have devolved a national intention for standards-based assessment to regional governments. Some states, such as Netherlands and Poland, have third party, voluntary accreditation, with varying incentives for participation.

This study has shown many opportunities for improving healthcare services by sharing experience of effective quality management, both within and between countries. Even without statutory or governmental enforcement, best practice and guidance from many sources could usefully be developed into a voluntary code of conduct for all hospitals in Europe. The measurement tool, piloted in this project, could be developed into a practical mechanism for voluntary self-assessment or peer review for organisational development of individual hospitals and to promote consistency of quality and safety for patients across European borders.
